# Allocating resources to support universal health coverage: policy processes and implementation in Malawi

**DOI:** 10.1136/bmjgh-2020-002766

**Published:** 2020-08-25

**Authors:** Pakwanja Twea, Gerald Manthalu, Sakshi Mohan

**Affiliations:** 1 Department of Planning and Policy Development, Ministry of Health, Lilongwe, Malawi; 2 Center for Health Economics (CHE), University of York, York, UK

**Keywords:** health policy, health economics

## Abstract

Optimising the use of limited health resources in low-income and middle-income countries towards the maximisation of health outcomes requires efficient distribution of resources across health services and geographical areas. While technical research exists on how efficiencies can be achieved in resource allocation, there is limited guidance on the policy processes required to convert these technical inputs into practicable solutions. In this article, we discuss Malawi’s experience in 2019 of revising its resource allocation formula (RAF) for the geographical distribution of the government health sector budget to the decentralised units in-charge of delivering primary and secondary healthcare. The policy process to revise the RAF in Malawi was initiated by district assemblies seeking a more equitable distribution of government resources, with the Ministry of Health and Population (MOHP) leading the technical and deliberative work. This article discusses all the steps undertaken by MOHP, Malawi to date as well as the steps necessary looking forward to legally establish the newly developed RAF and to start implementing it. We highlight the practical and political considerations in ensuring the acceptability and implementation feasibility of a revised RAF. It is hoped that this discussion will serve as guidance to other countries undergoing a revision of their resource allocation frameworks.

**Summary box**

- In the context of the growing decentralisation of the management and delivery of public services in low-income and middle-income countries, objective resource allocation formulae provide a mechanism to improve the efficiency, equity and political acceptability of the distribution of public funds to devolved units.
- While the decision on resource allocation is primarily a strategic and technical decision, practical and political considerations are equally important to ensure the effective implementation of the chosen resource allocation framework.
- Malawi’s experience of revising its health sector resource allocation formula in 2019. Demonstrates that following due process for the adoption and implementation of a formula is crucial.

## Introduction

The global movement towards the devolution of essential government functions has been accompanied by a quest for effective resource allocation frameworks.^1 2^ There has been a rising adoption of a formula-based approach to resource allocation among low and middle-income countries,^3 4^ with the objective of achieving efficient and equitable allocation across devolved units in-charge of delivering public services. There is growing literature on the principles of formula design,^5^ possible frameworks,^6–8^ as well as challenges to the implementation of a formula.^4^ Missing in the literature, however, is an illustration of the practical processes required to adopt and implement a new formula for resource allocation. Given that there is no gold standard for the components of an ‘ideal’ resource allocation formula (RAF),^6^ it is particularly important to follow a systematic process for arriving at a nationally accepted formula which adequately captures contextual determinants of service delivery costs. This paper attempts to fill this gap by describing Malawi’s recent experience of designing a new practicable RAF, based on the technical foundation presented in McGuire *et al* (2020)^8^ to distribute its health sector budget to districts. In this vein, the below can serve as a guide to other decentralised governments seeking to revise their RAFs.

## Context

In 2005, Malawi devolved the operational budgets of the health, education and agriculture sectors to district assemblies.^9^ For the health sector, this meant that the district health office (DHO) became responsible for the delivery of primary and secondary healthcare. Today, DHOs are assigned an independent recurrent budget, consisting of earmarked ceilings for drug, personnel emoluments (PE; salaries of staff) and other recurrent transactions (ORT); capital expenditure remains centralised.

For the allocation of this budget across districts, a formula was developed in 2002, and further revised in 2008. These formulae were based on a set of proxy indicators to represent the likely variation in health expenditures among districts. Due to limited follow-through, however, these formulas were eventually replaced by incremental historical allocation.

In 2018, through the initiative of the National Local Government Finance Committee (NLGFC)—the central decision-making body responsible for resource allocations to local government—triggered by a recognition by district assemblies of inequities in resource allocation,^10^ there was a push to better align intragovernmental fiscal transfers across sectors with regional needs. It was in the context of this political impetus that the Ministry of Health and Population (MOHP) began the technical work to revise the RAF for the health sector.

## Linking the resource allocation formula with the essential health package

Malawi has had a long history with health benefits packages (HBPs), called ‘Essential Health Packages (EHP)’, with its first officially recognised HBP being adopted in 2004.^11^ In 2017, a new HBP was adopted with the objective of tacking the persistent challenge of affordability among previous HBPs. The 2017 EHP was a positive list of 97 health services chosen under the guiding principles of health maximisation, cost-effectiveness, equity, continuum of care and complementarity of services, while allowing for exceptions for donor-funded health interventions.^12^

The primacy of the HBP coverage as a goal for the MOHP meant that the HBP served as a foundation for the design of the RAF.^8^ McGuire *et al* (2020) proposed four theoretical frameworks within which the formula for Malawi could be placed—ranging from the easy to implement and highly parsimonious framework based simply on the size of the population, on the one hand, to a more comprehensive and accurate framework based on the detailed calculation of expected service delivery cost based on population size, disease burden, unit cost of treatment and current coverage rates. The shape of the HBP, that is, the level of specificity of interventions included in the package, in Malawi, along with the availability of adequately granular epidemiological and treatment cost data, and the political preference for accuracy, implied that the more accurate ‘bottom-up epidemiological approaches’^13^ could be applied. Once the broad framework was chosen, additional determinants on cost variations across districts namely the number of operational secondary-care hospitals, dispersion of health facilities and staff shortage (addressed through temporary hires and overtime payments) were included in the final formula.

## Process of revising the resource allocation formula

**Figure 1:**
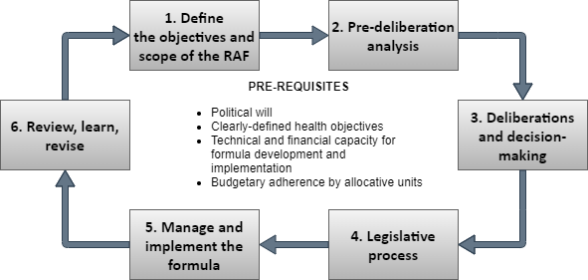
Process of revising a resource allocation formula. RAF, resource allocation formula.

Broadly, the process of adopting an RAF can be broken down into six steps (see figure 1). The first step involves defining the objective and scope of the RAF which provides the foundation for the design and subsequent adoption of a new RAF. Step 2 is analysing possible formula frameworks against four main criteria: (1) alignment with strategic objectives, (2) technical soundness, (3) implementation feasibility and (4) political acceptability. This analysis provides the groundwork to guide step 3 in the process, which is holding deliberations with relevant stakeholders to present and discuss formula options. These deliberations are intended to lead up to a decision at the technical level which is followed by step 4: undertaking the legislative process necessary to establish the legal mandate to implement the formula. Step 5 begins after the formula is adopted in practice. This step consists of constant oversight from the technical government institution (usually the Ministry of Health) to ensure the effective implementation of the formula. Finally, under step 6, a regular and institutionalised process of reviewing the formula is put in place in order to ensure the continued relevance, acceptance and implementation of the formula.

In this section, we present Malawi’s experience with carrying out the above process to revise the country’s RAF for the health sector in 2019. The precise nature and scope of each step will vary across countries based on the nature of organisation of their health systems and governance structures, but the overall process is expected to be similar to what is presented here.

### 1. Define the objectives and scope of the resource allocation formula

Objectives of formula-funding usually lie within the broad groups of efficiency, equity and political considerations. However, in order to operationalise these objectives into a formula, they need to be made more explicit.

The objectives of the RAF for Malawi were drawn directly from the Health Sector Strategic Plan II, a strategic document identifying national priorities for investments in the health sector between 2017 and 2022. The objective of efficiency was operationalised by aligning the formula-based allocation to the HBP which is rooted in the principles of cost-effectiveness and health maximisation. The second objective of equity was characterised as providing ‘equal opportunity to deliver the standard level of health services specified’^8^ in the HBP to all allocative units. Finally, like any other public health policy, the RAF was formulated within the context of the politics of the country, with the objective of improving the acceptability of resource distribution among all stakeholders.

In addition to the objectives of formula-based allocation, the scope of application of the formula also has significant implications for the RAF. Since donor resources no longer operate through a pooled fund managed by the government in Malawi,^14^ the formula could only be applied to the domestic health sector budget. Furthermore, within the government budget, the formula was only intended for drugs and ORT. The PE budget had to be excluded because this is determined by the distribution of the health workforce, currently managed by the Department of Human Resources and Development which is independent of the MOHP and district assemblies. Similarly, the development budget (for infrastructure expenses) was excluded because it is centrally managed by the MOHP. The scope of the formula also requires consideration of the level of the decentralised health system at which the formula will apply. In Malawi, the formula was developed to distribute resources to the 29 DHOs responsible for delivering primary and secondary healthcare through a network of health facilities. Allocations below this level were left to the discretion of the DHOs due to the lack of granularity of data available at this level.

### 2. Pre-deliberation analysis

Defining the objectives and scope of formula-based allocation sets the parameters within which the appropriateness of different formula frameworks can be evaluated. The proposed formula frameworks were evaluated against four criteria: (1) alignment with strategic objectives, (2) technical soundness, (3) implementation feasibility and (4) political acceptability.

Based on the first and second criteria, the first option proposed in McGuire *et al* (2020),^8^ based solely on population size was rejected since it did not account for the national objective of maximising health impact through the implementation of the cost-effective interventions in the HBP. Under the third criterion of implementation feasibility, the completeness, granularity, timeliness and reliability of data required for the components of the formula were evaluated. This led to the rejection of the third option, which required the input of coverage rates of all interventions in the HBP, for which Malawi did not have district-level data.

Once the data had been assessed and gathered, simulations were carried out of the actual effect of each formula framework under consideration on the budget of the allocative units, in the current and future fiscal years. This analysis was considered crucial to gauge the likely political acceptability of the proposed formula options.

### 3. Deliberations and decision-making

A formula designed without adequate engagement of affected stakeholders is unlikely to receive the political support necessary for its adoption. In Malawi, deliberations on the RAF included the MOHP, NLGFC, Ministry of Finance (MOF), Ministry of Local Government (MOLG), DHOs, District Councils and certain external partners engaged in project implementation at the district level. Consultations were held at two stages in the development of the RAF: first, to present broad frameworks and their budget implications, and gather ground perspective on key drivers of service delivery costs, and next, to present the more specific formula options, which incorporated suggested modifications from the first consultation, to reach consensus on measurement, weightage and data sources.

When presenting budget impact analyses, allocative units were anonymised. This helped ensure that the discussions were not biased by the inevitable creation of ‘winners’ and ‘losers’ under the revised allocation frameworks, that is, the discussion remained focused on the objectives and components of the formula and not its implications for individual districts. After the second consultation, the MOHP shared its final RAF proposal with all the relevant stakeholders for a final round of validation before finalising the RAF to be taken forward to the legislative body.

### 4. Legislative process

In most democratic countries, decisions made by the executive body are not legally enforceable. For transparency, legal enforceability, and stability (across transitional governments), adoption of the formula by the legislative body is crucial.

In Malawi, the Parliamentary Committee on Health, composed of a subset of the elected Members of Parliament, represents health sector issues in the Parliament. The first step, therefore, is to convince the Parliamentary Committee on Health of the merit of the revised RAF. Next, the Ministry of Justice to prepares draft bill/policy for the consideration of the Parliament. After the draft bill has been vetted by the MOHP, and other relevant ministries, such as the MOLG, the bill can be presented to the Parliament during its session. Timing these processes correctly is crucial for effective execution. Since parliamentary approval is required ahead of resource allocation decisions for the subsequent financial year, the MOHP is working to finalise the executive decision in 2020 so that the bill can be tabled during the March 2021 session of the Parliament, ahead of the financial year starting July 2021.

Since the legislative process can be expensive and time-consuming, efficiencies can be gained if the formulas for the other sectors are also finalised simultaneously with the health sector. In Malawi, the NLGFC is leading this process of intersectoral coordination in formula design.

### 5. Manage and implement the resource allocation formula

On legislative approval, implementation of the RAF can begin. If the formula implies significant redistribution, it is usually not advisable to transition to formula-based allocation within one fiscal year—both to avoid political backlash as well as to allow sufficient time for allocative units to absorb shocks to their budget. In Malawi, absolute budget cuts to any district are not politically feasible. This means that only the increase in the total resource envelope can be re-allocated in order to move the overall distribution closer to the *ideal* distribution of resources. In the context of a slow-growing government budget in Malawi as well as required outward reallocations as large as 50% of the current percentage allocation, this will imply a long phase-in period.

The long duration of phase-in necessitates regular communication between the MOHP and NLGFC to guarantee the sustained implementation of the RAF, in order to avoid the 2008 situation of the formula being abandoned after a few years of implementation.

It is equally important to ensure adherence to the assigned ceiling. In the past, districts have often overspent their drugs budgets by receiving additional funds from the MOF on request. While such exceptions can and should be made under emergencies, regularly exceeding budgetary limits will lead to formula funding remaining a mere ritual.^5^

Simultaneously, it is vital also for MOHP also to ensure that the formula responds in a timely manner to any significant changes to the cost of service delivery in the health sector, such as drug price revisions, changes in donor contributions, movement of sub-population groups and so on. Inadequate response to these changes can negatively influence efficiency and equity.

### 6. Review, learn, revise

The role of the MOHP does not end at the point of providing ‘equal opportunity’ to all allocative units to deliver services included in the HBP. Regular monitoring of how budgets are actually used is required. If the MOHP observes that the budget is not being spent on the HBP, other binding or non-binding measures^15^ may be adopted to nudge allocative units in the right direction. Monitoring budget performance can also throw light on any constraints or advantages particular to certain allocative units which the formula might not have considered. In Malawi, the annual Resource Mapping process will support such a regular performance analysis.

Given that Malawi’s health strategic plan, along with the HBP, is planned to be revised every 5 years, the review process for the RAF will also need to take place every 5 years at minimum. It was also agreed that any major updates to the inputs in the formula would be made as and when this information becomes available. For example, if new hospitals become operational, this should accordingly be updated in the formula, to ensure adequate allocation to the district with a new hospital.

## Conclusion

This article has provided a brief description of the steps being followed by the Government of Malawi to revise the formula for the allocation of health sector resources to devolved units. We have highlighted that political impetus from the appropriate channel of authority is essential to trigger the effort for the adoption and implementation of a revised RAF.

In terms of the design of the formula, a more comprehensive bottom–up epidemiological estimation of cost of service delivery could be adopted in Malawi due to the existence of an explicit HBP, as well as accompanying cost and epidemiological data which had been gathered during the development of the HBP. In countries where such an explicit HBP does not exist or where data at the required level of disaggregation is not available, a broader formula based on proxy indicators which adequately capture the variations in health needs of the relevant population sub-groups may be used to achieve the same objective.

Malawi has learnt from past experience that good design is not sufficient for the successful implementation of an RAF. Regular communication with the NLGFC, in-charge of allocation of district budgets, is essential especially in the context of high staff turnover and limitations in the knowledge transfer systems. It is equally important to recognise that formula-based allocation is only an instrument for creating sub-national financial equity and does not guarantee the alignment of health expenditure to public health priorities. It is, therefore, not a substitute for the monitoring of local health budget performance by MOHP.

This article has focused on using a RAF for an efficient distribution of government resources across semi-autonomous regions of a country. The reality of most low-income countries is, however, that a large proportion of health expenditure is borne by external financing sources. With more than half of the health expenditure accounted for by external partners^16^ in Malawi, ignoring the distribution of external resources is likely to limit the impact of the RAF on efficiency and equity. Additional measures may be required to coordinate disparate external partners towards optimal resource allocation.

We also highlighted that the goal of the RAF in Malawi was to provide ‘equal opportunity’ to all DHOs to deliver the EHP. In an ideal scenario, formula-based estimates could be used to guide the sectoral allocation required from the MOF so that districts would be allocated the full expected cost of service delivery. In reality, resources are often insufficient to meet the full need, often due to the adoption of unrealistic HBPs. This implies that there is usually an implicit rationing of services by healthcare providers.^17^ If the HBP is unrealistic, the RAF may also explicitly provide guidance on the rationing of services by basing allocation on the most cost-effective subset of services in the HBP. Low-income and middle-income countries face a further challenge of a mismatch between cash disbursements and budget allocation^4^ which creates a further constraint on effective planning.

Just as a sustainable HBP requires ‘constant review and revision, as new evidence emerges, new technologies are developed, and national circumstances evolve’,^15^ so too the RAF needs to accommodate changes to the HBP, as well as changes in the costs of treatment, demographic patterns and epidemiological patterns. Failure to update the RAF as circumstances change will cause the system to revert to a sub-optimal allocation of resources, misaligned with the public health objectives of the country.
